# Mesobiliverdin IXα Enhances Rat Pancreatic Islet Yield and Function

**DOI:** 10.3389/fphar.2013.00050

**Published:** 2013-04-23

**Authors:** Taihei Ito, Dong Chen, Cheng-Wei Tom Chang, Takashi Kenmochi, Tomonori Saito, Satoshi Suzuki, Jon Y. Takemoto

**Affiliations:** ^1^Department of Organ Transplant Surgery, School of Medicine, Fujita Health UniversityToyoake, Aichi, Japan; ^2^Synthetic Bioproducts Center, Utah State UniversityLogan, UT, USA; ^3^Department of Biological Engineering, Utah State UniversityLogan, UT, USA; ^4^Department of Chemistry and Biochemistry, Utah State UniversityLogan, UT, USA; ^5^Department of Clinical Research Center, Chiba-East National Hospital, National Hospital OrganizationChiba City, Chiba, Japan; ^6^Human and Animal Bridging Research Organization Research Laboratories, Ichikawa General HospitalIchikawa, Chiba, Japan; ^7^Department of Biology, Utah State UniversityLogan, UT, USA

**Keywords:** mesobiliverdin, biliverdin, pancreatic islets, islet transplantation, anti-inflammatory

## Abstract

The aims of this study were to produce mesobiliverdin IXα, an analog of anti-inflammatory biliverdin IXα, and to test its ability to enhance rat pancreatic islet yield for allograft transplantation into diabetic recipients. Mesobiliverdin IXα was synthesized from phycocyanobilin derived from cyanobacteria, and its identity and purity were analyzed by chromatographic and spectroscopic methods. Mesobiliverdin IXα was a substrate for human NADPH biliverdin reductase. Excised Lewis rat pancreata infused with mesobiliverdin IXα and biliverdin IXα-HCl (1–100 μM) yielded islet equivalents as high as 86.7 and 36.5%, respectively, above those from non-treated controls, and the islets showed a high degree of viability based on dithizone staining. When transplanted into livers of streptozotocin-induced diabetic rats, islets from pancreata infused with mesobiliverdin IXα lowered non-fasting blood glucose (BG) levels in 55.6% of the recipients and in 22.2% of control recipients. In intravenous glucose tolerance tests, fasting BG levels of 56 post-operative day recipients with islets from mesobiliverdin IXα infused pancreata were lower than those for controls and showed responses that indicate recovery of insulin-dependent function. In conclusion, mesobiliverdin IXα infusion of pancreata enhanced yields of functional islets capable of reversing insulin dysfunction in diabetic recipients. Since its production is scalable, mesobiliverdin IXα has clinical potential as a protectant of pancreatic islets for allograft transplantation.

## Introduction

**Figure 1:**
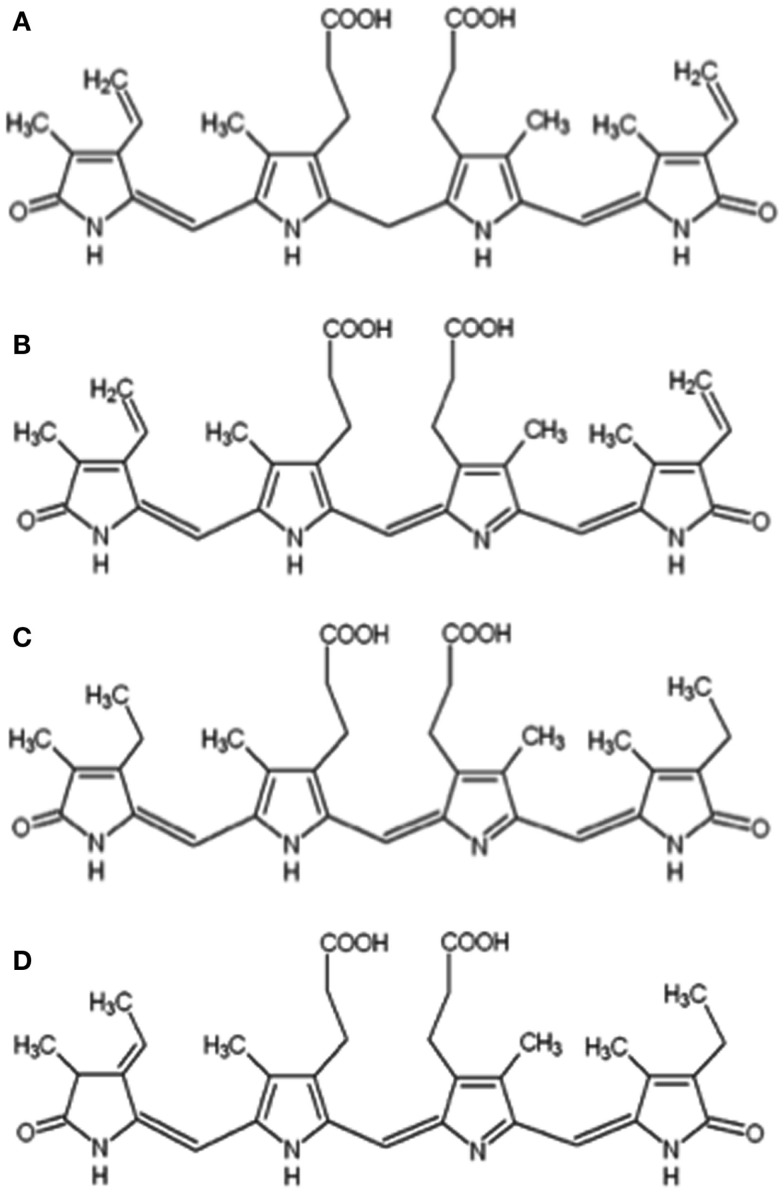
**Chemical structures of bilirubin IXα (A), biliverdin IXα (B), mesobiliverdin IXα (C), and phycocyanobilin (D)**.

The bile pigments bilirubin (Figure 1A) and biliverdin (Figure 1B) are best known as heme degradative intermediates associated with erythrocyte and hemoglobin turnover (McDonagh, 2001). They result from ring cleavage of heme catalyzed by heme oxygenase (HO) that occurs selectively at the α-methene bridge to generate the IXα isomer of biliverdin. Biliverdin IXα is subsequently reduced via NADPH/NADH biliverdin reductase to form the IXα isomer of bilirubin that in turn is consecutively bound to serum albumin and glucuronic acid for excretion in bile. The overall process serves to eliminate heme – which is toxic when accumulated.

Biliverdin IXα and bilirubin IXα are also cytoprotectants (Stocker et al., 1987; Sedlak and Snyder, 2004; Soares and Bach, 2009; Kapitulnik and Maines, 2012). Bilirubin IXα is well known to quench the propagation of reactive oxygen species (ROS) and consequently to confer protection against cellular oxidative damage. Biliverdin IXα is less appreciated as an anti-oxidant but equally effective (Stocker et al., 1987), and examples of its cytoprotective capabilities are accumulating (Nakao et al., 2004, 2005; Yamashita et al., 2004; Overhaus et al., 2006; Fujii et al., 2010; Bellner et al., 2011). The cytoprotective effects of biliverdin IXα also result from interaction with biliverdin reductase that plays a pivotal role in multiple downstream pathways related to cell survival and stress responses (Gibbs and Maines, 2007; Gibbs et al., 2012; Wegiel and Otterbein, 2012). Examples of biliverdin reductase mediated cytoprotective pathways are PI3K/Akt pathway-dependent protection against hypoxia/reoxygenation (Pachori et al., 2007), regulation of anti-apoptotic transcription factor NF-κB (Gibbs and Maines, 2007), induction of anti-inflammatory cytokine interferon-10 (Wegiel et al., 2009) and the nitrosylation-dependent inhibition of pro-inflammatory TLR4 expression (Wegiel and Otterbein, 2012). Thus, biliverdin IXα is increasingly recognized as a potential anti-inflammatory therapeutic agent (Florczyk et al., 2008; Wang et al., 2011; Gibbs et al., 2012; Wegiel and Otterbein, 2012). Examples of its potential use are for ischemia/reperfusion injury following liver (Fondevila et al., 2003; Nakao et al., 2004), small bowel (Nakao et al., 2004), cardiac, renal (Nakao et al., 2005), and lung (Zhou et al., 2011) transplants, vascular injury (Ollinger et al., 2005), endotoxic shock (Sarady-Andrews et al., 2005), vascular intimal hyperplasia (Nakao et al., 2005), nephropathy (Fujii et al., 2010), infection by hepatitis C (Zhu et al., 2010) and other viruses (Nakagami et al., 1992; McPhee et al., 1996), and reversal of type 2 diabetes by diets supplemented with biliverdin IXα (Ikeda et al., 2011). Barriers to the therapeutic use of biliverdin IXα are limited availability, uncertain purity of commercial preparations and derivation from mammalian materials (McDonagh, 2005) prompting attempts to substitute other bile pigments to achieve therapeutic effects (Zheng et al., 2012).

Another possible application for biliverdin IXα is improvement of pancreatic islet allograft transplantation efficacy (Najarian et al., 1977; Matsumoto et al., 2007). In this procedure, normal islets are excised from donor pancreata, preserved in solution, and injected into the intraportal ducts of type 1 diabetic recipients leading to insulin independence and hypoglycemia awareness. The procedure is historically hindered by allograft rejection and oxidative damage of islet beta cells. Immunosuppressive strategies have lowered islet rejection rates (Shapiro et al., 2000; Ryan et al., 2004; Matsumoto et al., 2007; Kenmochi et al., 2008), but the procedure is still hampered by oxidative-stress induced apoptosis that reduces the number of transplanted islets (Emamaullee and Shapiro, 2006; Wang et al., 2011). Anti-inflammatory strategies that improve the number of effective transplanted islets include stimulation of HO expression (Ribeiro et al., 2003), bilirubin IXα administration to recipient or donor islets during processing (Wang et al., 2011), and administration of p38 MAPK inhibitor to donor pancreata (Ito et al., 2008). Biliverdin IXα as an anti-inflammatory islet protectant has not yet been reported due at least partly to the limited amounts of commercially available biliverdin IXα.

Here we report the production of mesobiliverdin IXα (Figure 1C), a close analog of biliverdin IXα, and determination of its ability to protect islets. Mesobiliverdin IXα occurs naturally in non-vertebrates and mammals, and in the latter from bacterial transformations of non-conjugated bilirubin (Greenberg et al., 1971; Tiribelli and Ostrow, 2005; Vitek et al., 2006). Mesobiliverdin IXα and biliverdin IXα share important structural features (e.g., bridging propionate groups) that permit similar substrate interaction with biliverdin reductase (Cunningham et al., 2000; Fu et al., 2012) and suggesting similar cytoprotective capabilities against cellular damage by ROS. Importantly, the described method for mesobiliverdin IXα production is scalable and uses an abundant non-animal source feedstock – cyanobacteria. Finally, we show the abilities of the produced mesobiliverdin IXα as well as biliverdin IXα-HCl to protect pancreatic islet preparations for allograft transplantation.

## Materials and Methods

### Mesobiliverdin IXα

Mesobiliverdin IXα was produced from the phycocyanin chromophore, phycocyanobilin (Figure 1D), recovered from lyophilized powders of the cyanobacterium *Spirulina platensis*. Phycocyanin was obtained by adding 160 g of Spirulina powder (Bio-Alternatives, Oregon, USA) to 2 L water, shaking the mixture on a rotary shaker overnight (16 h) at 200 rpm and 37°C, and centrifuging (90 min, 1597 × *g*) the mixture at 4°C. The supernatant fluid was recovered and 530 g of (NH_4_)_2_SO_4_ was slowly added with stirring to give a 50% saturated solution. The solution was incubated in ice water for 30 min. After centrifugation (15971 × *g*, 30 min), the dark-blue phycocyanin was collected and washed with 700 mL methanol. The centrifugation and washing (with 300 mL methanol) was repeated four times. Phycocyanobilin was obtained by cleavage of thioether bonds between the bile pigment and phycocyanin apoprotein. Washed phycocyanin generated from 160 g Spirulina powder was added to 600 mL methanol and reflux boiled with stirring for 16 h. After centrifugation at 6371 × *g* for 5 min, the supernatant fluid containing phycocyanobilin was recovered and concentrated to ∼40 mL by rotary evaporation. The concentrated phycocyanobilin solution was mixed with 25 mL chloroform and the mixture added to and shaken with 200 mL purified water (previously acidified with 300 μL 0.5 N HCl) in a separatory funnel. Phycocyanobilin was recovered in the chloroform layer. The pigment extraction was repeated three times with 10 mL volumes of chloroform. The chloroform fractions were combined and reduced to ∼10 mL by evaporation with nitrogen gas. The reduced pigment solution was added to 60 mL hexane and centrifuged for 3 min at 4500 × *g* and the pigmented pellet was air-dried. Typical yields were ∼100 mg phycocyanobilin 160/g Spirulina powder. Phycocyanobilin (180 mg) was added to 40 ml methanol with 400 mg K_2_CO_3_ (10 mg/mL) and 400 mg NaHCO_3_. After boiling under reflux for 8 h, the solution was added to 200 mL water. Mesobiliverdin IXα was recovered by readjusting the pH to 4.0 followed by re-centrifugaton at 4500 × *g* for 5 min. The supernatant fluid was discarded and 20 mL H_2_O was added to wash the mesobiliverdin IXα pellet. The centrifugation and washing steps were repeated twice more. Mesobiliverdin IXα (160 mg) was obtained after freeze-drying (FreeZone Plus 4.5L Cascade Benchtop Freeze Dry System, Labconco, MO, USA).

### Biliverdin IXα

Biliverdin IXα-HCl was purchased from Frontier Scientific, Inc., Logan, UT (USA) and produced from recombinant *E. coli* (Chen et al., 2012).

### Analytical methods

Absorbance spectra were obtained using a SpectraMax Plus384 Absorbance Microplate Reader (Molecular Devices, Sunnyvale, CA, USA). Mesobiliverdin IXα samples (20 μL) were injected into an Alliance HPLC system (Waters, Manchester, UK) using a Symmetry^®^ C18 column (4.6 mm × 75 mm) and elution gradient with solvent A (99.9% H_2_0, 0.1% trifluoroacetic acid) and solvent B (99.9% methanol and 0.1% trifluoroacetic acid). The elution gradient program was: 100% solvent A, 1 min; 0–60% solvent B, 1 min; 60–100% solvent B, 8 min, 0–100% solvent A, 1 min; 100% solvent A, 4 min, with a flow rate of 1 ml/min. Proton NMR and two-dimensional COZY spectra of phycocyanobilin and mesobiliverdin IXα were collected on a Bruker AV400 with an inverse probe. For two-dimensional COZY experiments, 1024 × 256 data points were collected on F2 and F1, respectively, and the data were apodized with a Sinebell function and zero filled to 1K × 1K prior to Fourier transformation. Data were processed with Mnova NMR software (Mestrelab Research, Santiago de Compostela, Spain). For mass spectroscopy, samples were analyzed on a NanoACQUITY UPLC (Waters, Manchester, UK) and a Q-Tof Primer tandem mass spectrometer (Waters, Manchester, UK). Samples (3 μL) were introduced into a Symmetry^®^ C18 trapping column (180 μm × 20 mm) with NanoACQUITY Sample Manager (Waters, Manchester, UK) washed with 99% solvent A and 1% solvent B for 3 min at 15 μL/min. Solvent A was 99.9% H_2_0, 0.1% formic acid and solvent B was 99.9% acetonitrile and 0.1% formic acid. Chemicals were eluted from the trapping column over a BEH300 C4 column with a 70 min gradient (1% solvent B, 5 min; 1–50% solvent B, 15 min; 50–65% solvent B, 2 min; 65–85% solvent B, 21 min, 87% solvent B, 15 min, 87–1% solvent B, 3 min, and 1% solvent, 22 min) with flow rate 0.4 μL/min. Spectral scan time was 1.0 s.

### NADPH biliverdin reductase activity

The enzymatic conversion of mesobiliverdin IXα to mesobilirubin was measured using the Biliverdin Reductase Assay Kit (Sigma-Aldrich, St. Louis, MO, USA). One mg of mesobiliverdin IXα was dissolved in 2 mL methanol, and 0.2 mL was mixed with 1 mL of the kit assay buffer. The kit-supplied recombinant human biliverdin reductase A enzyme was suspended in 800 μL water, and 160 μL of the enzyme suspension was added to 480 μL of assay buffer. Assay buffer containing 200 μg/mL of mesobiliverdin IXα, *E. coli* produced biliverdin IXα or phycocyanobilin (50 μL), biliverdin reductase solution (200 μL), and NADPH solution (0.24 mg/mL NADPH in assay buffer, 750 μL) were combined and the absorbance spectrum between 300–800 nm was measured at 0, 15, 30, 45, 60, 90, 145, 240, and 360 min using a SpectraMax Plus384 Absorbance Microplate Reader (Molecular Devices, Sunnyvale, CA, USA).

### Pancreata treatment and islet equivalents

Male Lewis rats, 300–350 g, were purchased from Charles River Laboratories, Inc. (Japan). All rats were maintained in specific pathogen-free conditions of the animal care facility and handled in accordance with institutional guidelines of the Animal Care Committee of Chiba-East National Hospital, Japan. The pancreata from rats were procured 30 min after dissection of inferior vena cava as a warm ischemic injury. Islets were isolated and quantitated using described procedures (Ito et al., 2010). Briefly, pancreata were distended by the infusion of Hanks’ balanced salt solution supplemented with 0.1% bovine serum albumin (HBSS/BSA), 1 mg/mL of Liberase (Roche Diagnostics GmbH, Mannheim, Germany), and 1, 10, or 100 μM of commercial biliverdin IXα-HCl (Frontier Scientific, Inc., Logan, UT, USA), *E. coli* produced biliverdin IXα-HCl (Chen et al., 2012) or mesobiliverdin IXα. Control pancreata corresponding to each experimental test set with either commercial or *E. coli* produced biliverdin IXα-HCl or mesobiliverdin IXα were treated with the same solution mixture but with no bile pigment. The distended and treated pancreata were incubated at 37°C for 30 min. After incubation, ice-cold HBSS/BSA was added to stop enzymatic digestion. The pancreatic tissues were dissociated by repeated shaking and washing and islets were then purified by gradient centrifugation on Histopaque-1077 (Sigma-Aldrich, Japan) (Ito et al., 2010). The islets were then handpicked and the number of islets converted to the standard number of islet equivalents (IEQs) after dithizone staining (Hansen et al., 1989; Fiedor et al., 1996; Ching et al., 2001). Islet yields were expressed as IEQs/g pancreatic tissue. Photomicrographs of dithizone stained islets were obtained using a Nikon ECLIPSE TE2000-S microscope at x40 magnification. Average IEQ/g differences between groups were analyzed by the 2-tailed unpaired Student’s *t*-test and considered statistically significant when p values were <0.05.

### Islet transplantation and *in vivo* evaluation of engraftment islet function

Recipient Lewis rats were made diabetic with intravenous administration of streptozotocin (STZ, 70 mg kg^−1^) 7 days before transplantation. Diabetes was indicated by non-fasting blood glucose (BG) levels of >350 mg/dL in two consecutive measurements. Islets (∼500 IEQs) isolated from donor pancreata with 30 min warm ischemia treated with or without mesobiliverdin IXα were infused into the portal vein of a diabetic recipient rat using a 1 mL-capacity syringe with 25-gage winged needle under general anesthesia. Non-fasting BG levels were measured every 2 days before and after transplantation to monitor the engraftment of islets. Reversal of diabetes was indicated when BG levels of <200 mg/dL were determined in two consecutive measurements. To evaluate the effect of mesobiliverdin IXα on transplanted islet function, intravenous glucose tolerance tests were performed 56 post-operative days after transplantation. Under general anesthesia, test and control recipient rats were intravenously injected with 1 mL/kg of 50% (wt/vol) glucose, and BG levels were determined at 0, 2, 5, 10, 20, 30, and 45 min intervals.

## Results

### Mesobiliverdin IXα production and identification

**Figure 2:**
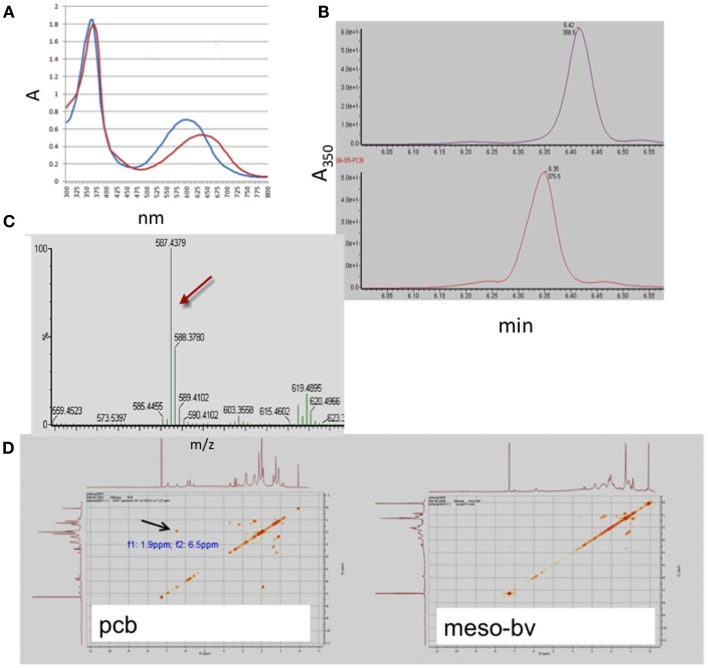
**Identification and structural analysis of mesobiliverdin IXα derived from phycocyanobilin**. Analyses performed were absorbance spectroscopy **(A)** of mesobiliverdin IXα (red) and phycocyanobilin (blue), HPLC **(B)** of mesobiliverdin IXα (top panel) and phycocyanobilin (bottom panel), mass spectroscopy **(C)** of mesobiliverdin IXα, and two-dimensional NMR COZY spectra **(D)** of mesobiliverdin IXα (mesoBV, right panel) and phycocyanobilin (pcb, left panel). The red arrow in **(C)** indicates a molecular mass of 587.4 for mesobiliverdin IXα. The black arrow in **(D)** indicates a = CH-CH3 functional group (at *f*_1_: 9 ppm; *f*_2_:6.5 ppm) in phycocyanobilin that is absent in mesobiliverdin IXα.

Mesobiliverdin IXα (Figure 1C) was produced by NaHCO_3_-K_2_CO_3_ – dependent isomerization of phycocyanobilin (Figure 1D) that in turn was derived and purified from dried preparations of the cyanobacterium *S. platensis* (Spirulina powder). Its identity and purity were determined by absorbance spectroscopy, TOF-ESI mass spectra, and two-dimensional NMR COZY analyses (Figure 2). Upon reaction with K_2_CO_3_ and NaHCO_3_, the phycocyanobilin 600 nm absorbance peak shifted to 640 nm and a 420 nm peak emerged (Figure 2A) indicating formation of a biliverdin-like compound. The HPLC retention time of the product was 0.07 min longer than phycocyanobilin (Figure 2B). In the phycocyanobilin two-dimensional NMR COZY spectrum, there were characteristic = CH-CH3 COZY correlations at 6.5 and 1.9 ppm that were absent in the product spectrum (Figure 2D) indicating the conversion of phycocyanobilin to mesobiliverdin IXα. Its molecular mass (587.4) (Figure 2C) confirmed the identity as mesobiliverdin IXα and occurrence in the free acid form. Its “IXα” analog designation was based on structural similarities to biliverdin IXα and specifically the replacement of ethyl groups in place of vinyl groups in the terminal pyrrole rings of biliverdin IXα. The mesobiliverdin IXα product was >90% pure as judged by HPLC (Figure 2B) and mass spectroscopy (Figure 2C). The latter also revealed small amounts of contaminant material with molecular mass 619.5 judged to be a phycocyanobilin-methanol adduct (Beuhler et al., 1976). Yields of purified mesobiliverdin IXα were linearly scalable at the rate of ∼100 mg/160 g of dry Spirulina powder.

### Mesobiliverdin IXα as substrate for NADPH biliverdin reductase

**Figure 3:**
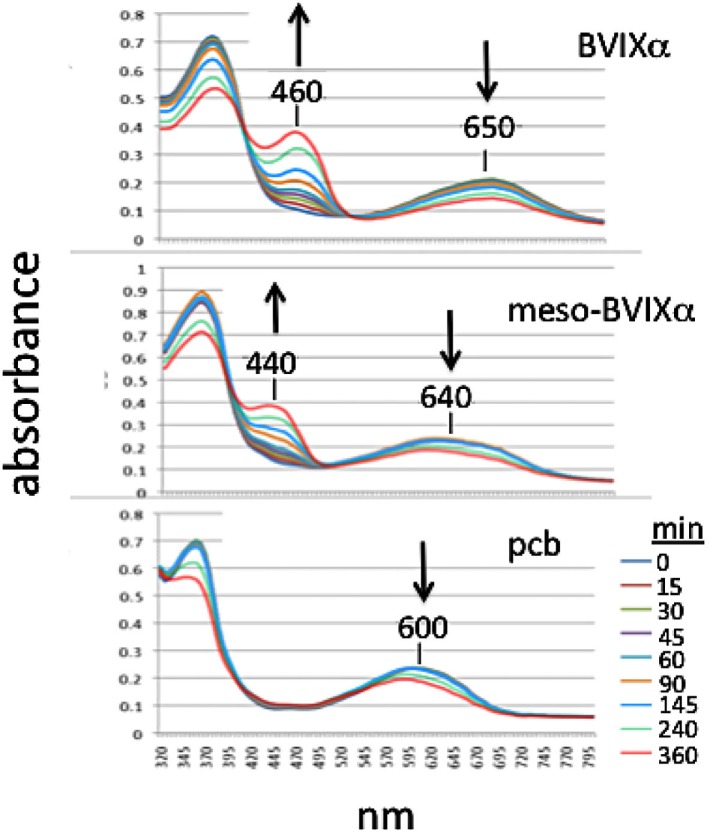
**Time-course of reactions catalyzed by human recombinant NADPH biliverdin reductase with *E. coli* produced biliverdin IXα-HCl (BVIXα), mesobiliverdin IXα (meso-BVIXα), and phycocyanobilin (pcb) as substrates**. NADPH-dependent reduction was monitored spectrophotometrically for 6 h.

As substrate for recombinant human NADPH bilirubin reductase, mesobiliverdin IXα was reduced to mesobilirubin [λ_max_, 440 nm (Terry et al., 1993)] at rates that were equivalent to those for catalytic conversion of biliverdin IXα to bilirubin IXα (λ_max_, 460 nm) (Figure 3). In contrast, phycocyanobilin, the synthetic precursor to mesobiliverdin IXα, was a relatively poor substrate as judged by the inability to detect catalytic conversion to phycocyanorubin [λ_max_, 420 nm (Terry et al., 1993)].

### Effect on pancreatic islet yield and viability

**Figure 4:**
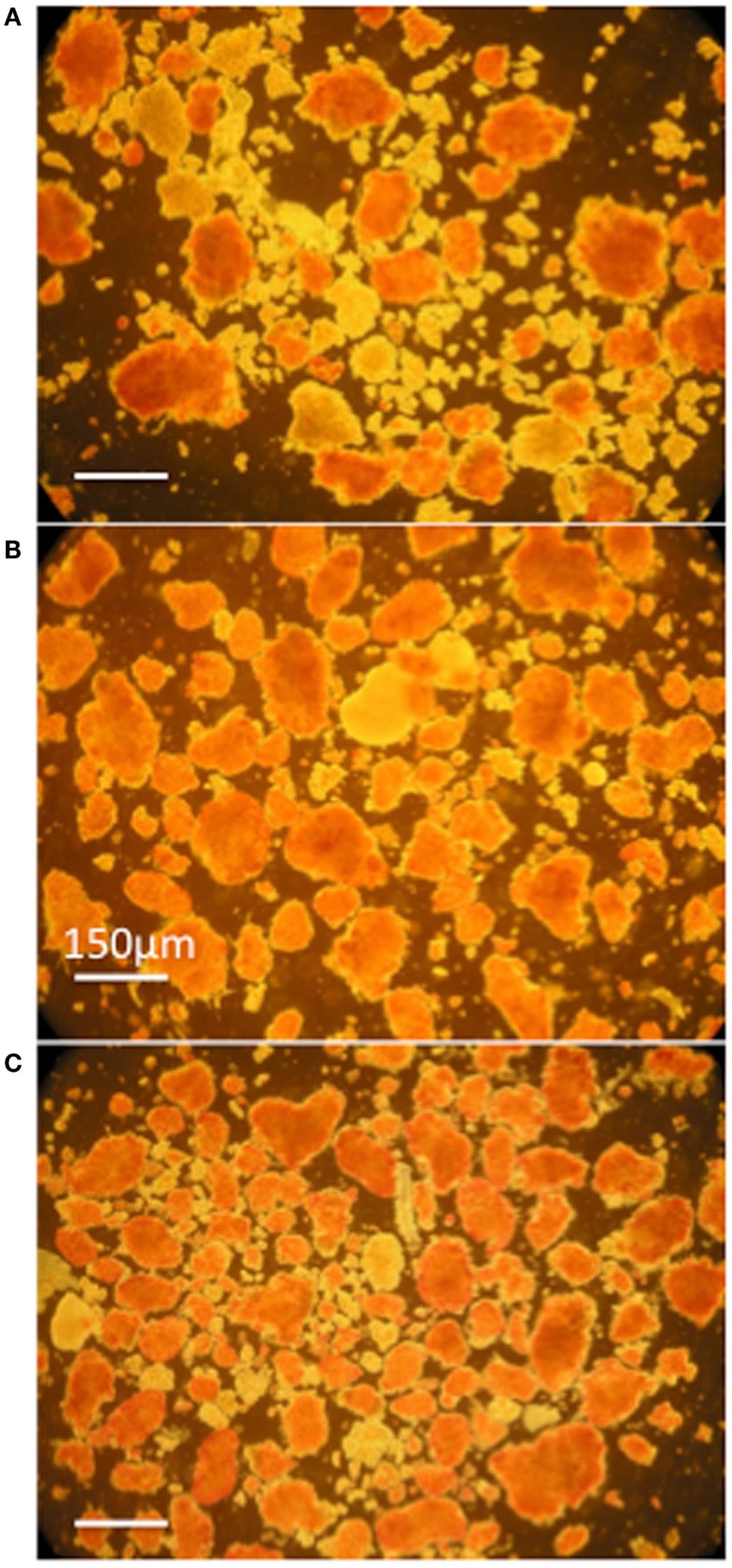
**Photomicrographs of dithizone stained islet preparations derived from donor Lewis rat pancreata infused with HBSS/BSA solution (A) and HBSS/BSA solution containing 100 μM mesobiliverdin IXα (B) or 100 μM *E. coli* produced biliverdin IXα-HCl (C)**. Viable islets are stained red. The bar designates a length of 150 μm.

Pancreata were infused with HBSS/BSA solutions containing mesobiliverdin IXα, commercial biliverdin IXα-HCl or *E. coli* produced biliverdin IXα-HCl, followed by islet isolation and determination of IEQs/g yields. Solutions containing mesobiliverdin IXα (at 1–100 μM) yielded IEQ/g increases ranging between 54 and 86.7% over controls (without mesobiliverdin IXα) (Table 1). The highest average IEQ/g (86.7% over controls) was achieved with 1 μM mesobiliverdin IXα. Infusion with solutions containing commercial biliverdin IXα-HCl gave IEQ/g average increases as high as 35.5% (at 10 μM) over controls and with recombinant *E. coli* produced biliverdin IXα-HCl, as high as 36.5% (at 100 μM). High degrees of islet viabilities as judged by dithizone staining were observed with mesobiliverdin IXα and *E. coli* produced biliverdin IXα-HCl and a comparatively lower degree of viability was observed with no treatment (Figure 4).

**Table 1:** **Islet yields from pancreata infused with biliverdin IXα-HCl and mesobiliverdin IXα**.

Treatment^1^	IEQs g^−1^ (average, std, range, no. of values)	*P* value	% above control
1 μM BV_FS_^2^	1328 ± 358 (591–1705) (8)	0.426	11.3
10 μM BV_FS_	1617 ± 451 (1006–2519) (8)	0.037	35.5
100 μM BV_FS_	1527 ± 403 (942–2363) (9)	0.050	28.0
Control	1193 ± 223 (931–1307) (9)		
1 μM BV_EC_^3^	1345 ± 629 (662–2234) (7)	0.860	4.3
10 μM BV_EC_	1603 ± 1073 (901–4117) (8)	0.480	24.4
100 μM BV_EC_	1759 ± 703 (658–2593) (8)	0.163	36.5
Control	1289 ± 559 (579–2182) (8)		
1 μM mesoBV^4^	1599 ± 475 (1004–2053) (7)	0.005	86.7
10 μM mesoBV	1318 ± 805 (655–2946) (8)	0.156	54.0
100 μM mesoBV	1535 ± 287 (1145–1923) (8)	0.0002	79.3
Control	856 ± 229 (539–1166) (8)		
20 μM p38IH^5^	2134 ± 297 (997–2837)	0.037	45.1
Control^5^	1477 ± 145 (1118–1889)	0.037	

*^1^Seven to nine organs per infusion treatment*.

*^2^Biliverdin IXα-HCl purchased from Frontier Scientific, Inc., Logan, UT, USA*.

*^3^Biliverdin IXα-HCl produced by recombinant *E. coli* as previously described (Chen et al., 2012)*.

*^4^Mesobiliverdin IXα (this work)*.

*^5^*p*38 MAPkinase inhibitor; data from canine experiments (six organs per infusion treatment) reported in Ito et al. (2008)*.

### Recipient *in vivo* islet function after treatment of donor pancreata with mesobiliverdin IXα

**Figure 5:**
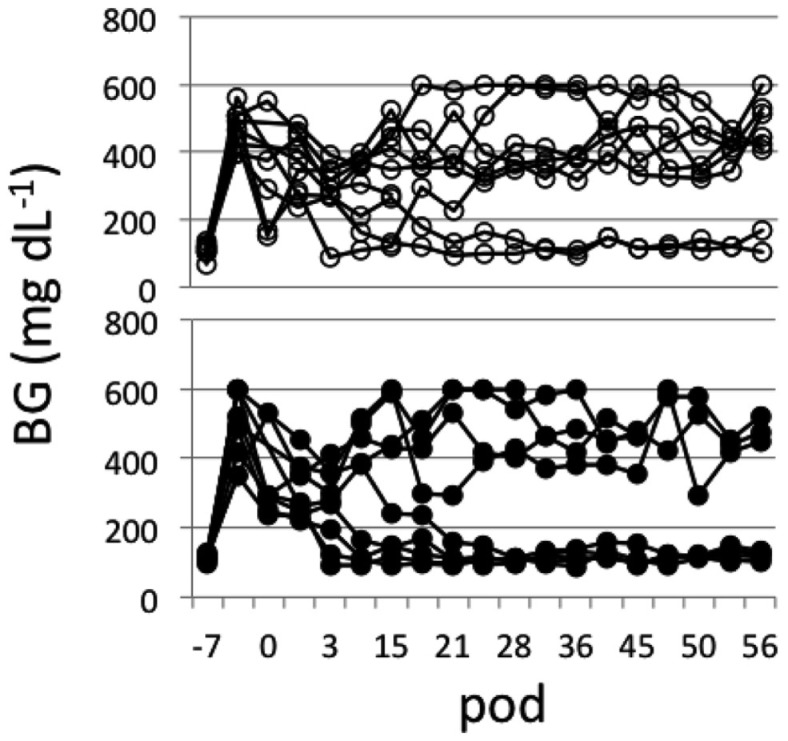
**Non-fasting blood glucose (BG) level profiles of STZ induced diabetic Lewis rats measured for up to 56 post-operative days (pod) following transplantation with islets (∼500 IEQs) from donor pancreata infused with HBSS/BSA solution containing 100 μM mesobiliverdin IXα (*n* = 9) (lower panel) or without mesobiliverdin IXα (*n* = 9) (upper panel)**.

**Figure 6:**
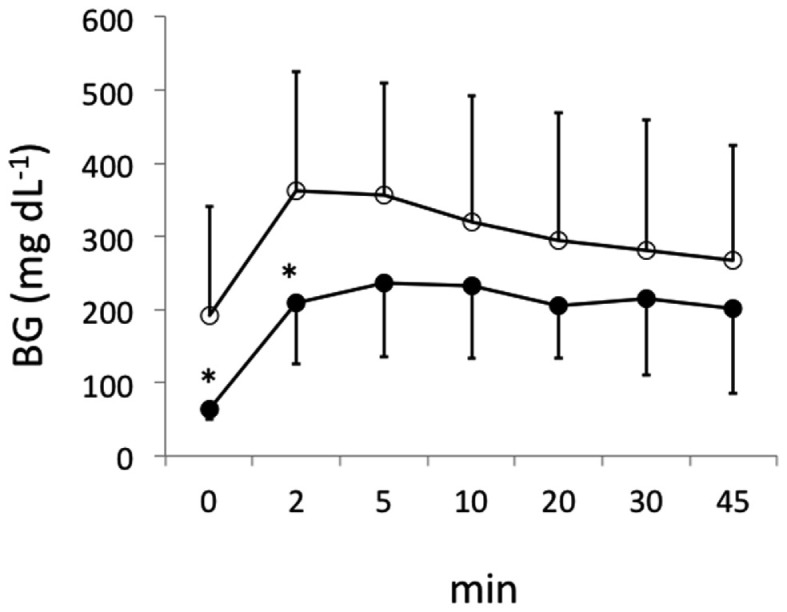
**Fasting blood glucose (BG) levels following intravenous tolerance tests of STZ induced diabetic Lewis rats**. Tests were performed on day 56 following transplantation with islets from donor pancreata infused with HBSS/BSA solution containing 100 μM mesobiliverdin IXα (*n* = 7) (filled circles) or without mesobiliverdin IXα (*n* = 9) (open circles). BG levels at 0 and 2 min correlated with first-phase insulin function. *p values < 0.05.

Islets (∼500 IEQs) obtained as described above with or without mesobiliverdin IXα (100 μM) treatment were transplanted by infusion into recipient livers of STZ induced diabetic rats through the portal vein. Subsequent recipient BG levels revealed diabetes reversal in 55.6% (five of nine) of the rats receiving islets from mesobiliverdin IXα 100 μM) – treated pancreata (Figure 5 lower panel); 22.2% (two of nine) of the non-treated control recipients showed diabetes reversal (Figure 5 upper panel). Intravenous glucose tolerance tests on day 56 also revealed improved islet function with mesobiliverdin IXα infusion of donor pancreata. Fasting BG levels measured at zero and 2 min indicated recovery of insulin-dependent function and were significantly lower with transplanted islets from pancreata treated with mesobiliverdin IXα as compared to controls with islets from non-treated pancreata (Figure 6).

## Discussion

Growing evidence suggests the therapeutic potential of biliverdin IXα against acute and chronic inflammatory conditions such as diabetes (Florczyk et al., 2008; Wang et al., 2011; Gibbs et al., 2012; Wegiel and Otterbein, 2012). Its current development as an anti-inflammatory pharmaceutical, however, is hampered by its commercial scarcity, contamination by isomers, and derivation from animal sources. The recently reported scalable production of biliverdin IXα by *E. coli* represents an attempt to address these issues (Chen et al., 2012). With the goal of producing a pharmaceutical equivalent of biliverdin IXα that also overcomes these limitations, the production of the mesobiliverdin IXα from a non-animal source (i.e., cyanobacteria) was developed. The synthesis of mesobiliverdin IXα from phycocyanobilin facilitated by NaHCO_3_-K_2_CO_3_ is efficient, as is the methanolic cleavage of phycocyanobilin from the apoprotein of phycocyanin. Phycocyanin itself is an abundant, water-soluble pigment-protein complex that in turn is easily extracted from photosynthetic microbes of the groups cyanobacteria, rhodophyta, and cryptophyta. Hence, the procedure is scalable for the production of large quantities of mesobiliverdin IXα.

Mesobiliverdin IXα differs from biliverdin IXα by the replacement of vinyl groups with ethyl groups at positions 3 and 18 of the linear tetrapyrrole structure (Figure 1). The differences are not expected to significantly affect substrate specificity binding to the active site of human biliverdin reductase for catalytic reduction to the corresponding product (i.e., mesobilirubin or bilirubin IXα) (Cunningham et al., 2000; Fu et al., 2012) as supported in the present study (Figure 3). Substrate binding to biliverdin reductase appears central to the downstream anti-inflammatory and anti-pro-inflammatory effects of biliverdin IXα (Gibbs et al., 2012; Wegiel and Otterbein, 2012). These considerations suggest that mesobiliverdin IXα may have therapeutic effects similar to those shown experimentally for biliverdin IXα in numerous animal studies. In comparison to mesobiliverdin IXα and biliverdin IXα, phycocyanobilin appeared to be a weaker substrate for NADPH biliverdin reductase (Figure 3) suggesting that it may have less effective anti-inflammatory capabilities (Zheng et al., 2012).

Both biliverdin IXα and mesobiliverdin IXα had protective effects against islet degradation following pancreatectomy. The degree of protection by mesobiliverdin IXα exceeded those provided by biliverdin IXα (Table 1). Pancreatic infusion with as low as 1 μM mesobiliverdin IXα gave nearly twofold higher IEQs/g than 10 and 100 μM biliverdin IXα and p38 MAPkinase inhibitor previously observed in canine islet transplant experiments (Table 1) (Ito et al., 2008). Mesobiliverdin IXα at 1 μM gave an average 86.7% increase in IEQs/g over non-treatment controls. This degree of improvement in islet yield is clinically significant since currently two or more pancreatic donor organs are required per recipient to achieve insulin independence (Shapiro et al., 2005; Ito et al., 2008; Wang et al., 2011).

In STZ induced diabetic rat transplantation experiments, mesobiliverdin IXα infusion with 30 min warm ischemic injury improved graft function of rat islets (Figures 5 and 6). Changes in fasting BG levels at 0 and 2 min in intravenous glucose tolerance tests indicated recovery of insulin-dependent function against a glucose load, and the overall levels were significantly lower than controls receiving islets from untreated pancreata. Thus improved yields of functional islets were achieved with mesobiliverdin IXα infusion of donor pancreata.

Why mesobiliverdin IXα at lower concentration (1 μM) protected better than commercial biliverdin IXα and *E. coli* produced biliverdin IXα is not known. Reasons may lie in differences of their chemical state and purity. The biliverdin IXα preparations used were of the hydrochloride form whereas the mesobiliverdin IXα was produced as the free acid – differences that could have bearing on tissue and cell accessibility. Commercial biliverdin IXα preparations derived from animal sources and produced from conjugated bilirubin often contain inactive isomers (McDonagh, 2005). The *E. coli* derived biliverdin IXα could possibly contain lipopolysaccharide endotoxin that would compromise its anti-inflammatory capabilities. Alternatively, the more cytoprotective effect of mesobiliverdin IXα may result from as yet unknown variations of the anti-inflammatory mechanisms of this bioactive porphyrin. Further investigations are needed to better understand the cytoprotective mechanisms of mesobiliverdin IXα in comparison to those for biliverdin IXα and other anti-inflammatory heme derived porphyrins.

## Conflict of Interest Statement

The authors declare that the research was conducted in the absence of any commercial or financial relationships that could be construed as a potential conflict of interest.
